# Clinical context and communication in shared decision-making about major surgery: Findings from a qualitative study with colorectal, orthopaedic and cardiac patients

**DOI:** 10.1177/13634593241238857

**Published:** 2024-03-21

**Authors:** Gemma Hughes, Timothy J Stephens, Lucas M Seuren, Rupert M Pearse, Sara E Shaw

**Affiliations:** University of Leicester, UK; University of Oxford, UK; Queen Mary University of London, UK; University of Oxford, UK; Queen Mary University of London, UK; University of Oxford, UK

**Keywords:** genre, linguistic ethnography, person-centred care, shared decision-making, surgery

## Abstract

Increasing numbers of older people undergo major surgery in the United Kingdom (UK), with many at high risk of complications due to age, co-morbidities or frailty. This article reports on a study of such patients and their clinicians engaged in shared decision-making. Shared decision-making is a collaborative approach that seeks to value and centre patients’ preferences, potentially addressing asymmetries of knowledge and power between clinicians and patients by countering medical authority with greater patient empowerment. We studied shared decision-making practices in the context of major surgery by recruiting 16 patients contemplating either colorectal, cardiac or joint replacement surgery in the UK National Health Service (NHS). Over 18 months 2019–2020, we observed and video-recorded decision-making consultations, studied the organisational and clinical context for consultations, and interviewed patients and clinicians about their experiences of making decisions. Linguistic ethnography, the study of communication and interaction in context, guided us to analyse the interplay between interactions (during consultations between clinicians, patients and family members) and clinical and organisational features of the contexts for those interactions. We found that the framing of consultations as being about life-saving or life-enhancing procedures was important in producing three different genres of consultations focused variously on: resolving problems, deliberation of options and evaluation of benefits of surgery. We conclude that medical authority persists, but can be used to create more deliberative opportunities for decision-making through amending the context for consultations in addition to adopting appropriate communication practices during surgical consultations.

## Introduction

Shared decision-making is part of the social context of changing relations between doctors and patients in western medicine which have ostensibly shifted during the 20th century from traditional, paternalistic, doctor-knows-best consultations to 21st century person-centred care that values the knowledge and preferences of patients (Buetow et al., 2009). This shift has not been without tensions. Detailed studies of patient-clinician encounters have shown how medical authority persists despite aspirations for greater patient empowerment (Stivers and Timmermans, 2020; Vinson, 2016). Notwithstanding these tensions, shared decision-making is generally regarded as an ethical way of taking patients’ views seriously when selecting courses of treatment. It is considered to be particularly important for patients considering major surgery who are at high risk of complications due to age and multi-morbidities. Their decisions about surgery are ‘high stakes’ (the decision to have surgery could lead to serious complications including vastly reduced quality of life and independence) and involve a high degree of decision conflict (uncertainty because treatment options do not have clear or predictably advantageous outcomes) (Boss et al., 2016). Yet relatively little is known about how such patients engage in shared decision-making (Shinkunas et al., 2020).

In this article we use linguistic ethnography, the study of communication and interaction in context (Tusting, 2019), to ask: how do patients regarded as high risk and their clinicians engage in shared decision-making in the context of major surgery? We argue for the importance of clinical and organisational context in producing different kinds of shared decision-making consultations. The *framing* of consultations (what they were understood to be about) shaped the interactions during those consultations, leading to varied opportunities for shared decision-making. These opportunities were made possible through, rather than by reducing, medical authority. The contribution of this article to the literature is a new analysis of how context shapes different kinds of shared decision-making, showing some of the ways in which tensions arise between the promise of shared decision-making to empower patients and its practice.

In the remainder of this article, we first provide an overview of debates about shared decision- making before setting out our methodological approach and empirical data. We then present our findings about how the varying clinical and organisational contexts and frames shaped different genres of decision-making consultations. Finally, we discuss these findings in relation to the extent to which opportunities for shared decision making are possible, how medical authority features in those opportunities and the implications for patient autonomy and choice.

## Background of shared decision making

### The potential good of shared decision-making

Shared decision-making has become a normative approach in 21st century healthcare for improving decision quality, reducing decision conflict, limiting variation in healthcare outcomes and reducing use of ‘low value’ procedures) (Boss et al., 2016; Santhirapala et al., 2019). Seen as essentially *good* (morally, in terms of medical practice, patient experience and outcomes), shared decision-making is incorporated into professional standards for doctors in the United Kingdom (UK) (General Medical Council, 2020). Shared decision-making is intended to produce ethical practice by upholding patient autonomy (the moral right to self-determination), one of the four principles of medical ethics (the others being beneficence, non-maleficence and justice) (Stiggelbout et al., 2015). By supporting patients to decide on treatment based on what matters to them, shared decision-making aligns closely with, and indeed is considered an important indicator of, patient-centred care (Coulter and Oldham, 2016; Pilnick, 2022).

Decisions about surgery, the focus of this article, involve irreversible courses of treatment with inherent risks. In addition to general risks of surgery, medical complications can lead to long-term problems for high-risk patients. The additional risks are related to comorbidities which are more likely, but not exclusively found, in older patients (Abbott et al., 2017; Fowler et al., 2022; Khuri et al., 1998; Pearse et al., 2012). Decisions to have surgery could, particularly for high-risk patients, lead to worse health. This problem, combined with a general lack of awareness about outcomes and difficulties in predicting them, has been the driver behind the research programme *Optimising Shared decision making for High Risk Major* Surgery (OSIRIS) of which the study reported in this article is part. Although shared decision-making could reduce decision conflict for high-risk patients, surgeons have been found to focus on helping patients who want ‘something done’ rather than ‘true’ shared decision-making (De Roo et al., 2021). Decisions to have surgery can be interpreted as choosing life over death, making it difficult to divert the clinical momentum towards surgery (Nabozny et al., 2016).

### Interactional models of shared decision-making

Models of shared decision-making have evolved to improve patient-clinician relationships whilst incorporating the complexities of collaborative decision-making and the ways in which is it socially situated and performed (Charles et al., 1997, 1999; Elwyn et al., 2014; Emanuel and Emanuel, 1992; Epstein and Street, 2011). A proportion of the literature on shared decision-making has centred on communication practices during patient-clinician encounters, drawing on a long tradition of studying communication in consultations (see e.g. Atkinson, 1995; Byrne and Long, 1976; Heritage and Maynard, 2005). The use of specific phrases and the structuring of the clinical encounter, for example through the use of option-listing, have been shown to shape the extent to which both clinicians’ authority is upheld and patients’ autonomy affirmed (Bélanger et al., 2016; Charles et al., 1999; Toerien et al., 2018). Improving interactions during clinical encounters, through clinical training, decision-support tools and patient activation, therefore appears to offer a route to enhanced shared decision-making.

### Shared decision making as a site of conflict

A more critical approach considers shared decision-making as enacted and situated, temporally and spatially distributed across social relations (Clapp et al., 2019; Pilnick and Zayts, 2016; Rapley, 2008). From this perspective, shared decision-making is shaped by, and contributes to, the power dynamics between doctors and patients, institutions and individuals. The ‘inherent asymmetry’ of doctor and patient relationships (Pilnick and Dingwall, 2011), involving epistemic injustices (Thomas et al., 2020), can therefore render shared decision-making a site of conflict between medical authority and patient empowerment. Medical authority is derived from both medical knowledge (epistemic domain) and the right to determine future actions such as making treatment recommendations (deontic domain). Interactional practices observed during patient-clinician encounters can involve resistance on the part of patients to medical authority, countered by persuasion from clinicians (Stivers and Timmermans, 2020). Despite the ostensive increase in patient empowerment supposedly brought about as a result of shared decision-making, collaboration can be constrained in the face of professional dominance (Vinson, 2016), with shared decision-making yet to prove a consistent solution to the conflict between medical authority and patient empowerment.

### Autonomy and agency

The normative model of shared decision-making as empowering patients and promoting their autonomy is further troubled as the dynamics of choice, dependency and trust between patients and clinicians play out in different settings (Borgstrom, 2015; Brown and Meyer, 2015), raising questions as to the extent to which patients want, and are able, to engage in decision-making. Many people express desire for autonomy in healthcare decisions (Cullati et al., 2011), but some prefer to leave decisions to their doctors (Levinson et al., 2005). Tensions arise between the person-centred principle of supporting patients to be autonomous decision-makers and the potential of adding to their burden of treatment by requiring them to take on responsibility for decision-making (Munthe et al., 2012). Patients might instead reasonably expect and want to trust their expert clinicians (whilst acknowledging their implicit positions of power) to take leadership in deciding on treatments (Schei, 2006).

Patients’ ability to exercise their agency and make choices can be constrained by social structures which curtail the social, economic and cultural power of specific groups of patients (Davies and Elwyn, 2008). However, it is the tension between the positions of clinicians and patients in terms of power and knowledge that can lead to individual autonomy appearing rather illusory; patients can feel they have no choice but to consent to, or go along with, recommended treatments (Dixon-Woods et al., 2006a; Polak and Green, 2020). This form of situated autonomy can be considered as more relational than self-determined, with decisions produced collectively from negotiations of inherently asymmetrical doctor-patient relationships (MacArtney et al., 2020).

In sum, the background of changing social norms about healthcare position shared decision-making as a moral good, obliging clinicians to fully engage all patients in shared decision- making, with the specific potential for increased knowledge for patients (about likely negative outcomes) and for clinicians (about patients’ preferences) that can reduce decision conflict and regret for high-risk patients contemplating major surgery. However, there remain tensions between the extent to which clinicians can employ communication practices to offer patients autonomy and choice during consultations and the social context of imbalances in power and knowledge between patients and clinicians. This article provides an analysis of such communication practices in context.

### Methodological approach

We set out to understand how patients regarded as high risk and their clinicians engaged in shared decision-making in the context of major surgery by locating shared decision making as part of the social organisation of healthcare work (Allen and Pilnick, 2005; Strauss, 1985). Our methodological approach was informed by linguistic ethnography (Tusting, 2019), guiding us to use a combination of ethnographic methods and interactional approaches to understand both organisational and clinical contexts and decision-making interactions. We describe below the key concepts informing the study and our analysis.

We approached decision-making as interactional work done by patients, clinicians and others as part of a patient’s illness *trajectory.* The sociological concept of patient trajectory refers to the unfolding events related to the course of a patient’s illness and the work involved in seeking help, undergoing assessment and diagnosis, and considering courses of action (Allen et al., 2004). All of the high-risk patients in our study had experienced long trajectories that, for many, had included previous experiences of major surgery. From observations and interviews (see Methods and research setting), we found certain features of patients’ trajectories were particularly important in shaping their consultations: the condition which led the patient to consider surgery, how that condition was understood by patient and clinicians, the organisation of services to respond to that condition, and patients’ co-morbidities. Our understanding of context was therefore as dynamic and relational (following Meier and Dopson, 2021), producing and produced by the actions of individuals and organisational routines concerned with decision-making processes.

We followed Huisman’s (2001) definition of a decision as talk that made ‘commitment to future action’, in this case talk between patients, families and clinicians that enabled a course of action to be agreed in relation to the proposition of major surgery. Our conceptualisation of a decision was therefore as an interactional accomplishment between participants rather than a cognitive process.

We used the concept of genre as a methodological device to analyse connections between interactions during consultations and the clinical and organisational context for those consultations. Genre explains how interaction between context and language operates in established ways to achieve certain functions. A genre is characterised by substance (social motives, themes and topics) and recognisable form (physical and linguistic features of the communication) (Blum-Kulka, 2004; Yates and Orlikowski, 1992, 2002). For example, a job interview is a genre of interaction that includes recognisable forms of communication as part of a wider system of recruitment practices for the purpose of hiring someone to do a job (Yates and Orlikowski, 2002). In our study, the communication practices that took place during consultations were part of a wider system of healthcare practices. Genres of consultation varied across, and in some cases during, consultations and surgical specialities but had common features and distinct purposes. The framing of consultations (e.g. as being about life-saving surgery) was particularly important in shaping how consultations were approached by patients and clinicians. Framing, from Goffman’s (1986) work, explains how social frameworks provide the background for how people might interpret and act within a particular social or institutional setting. Frames are both created by different institutional and social contexts and, in our study, shaped how patients and clinicians interacted in those differing contexts to produce different genres of consultations. In our analysis, we therefore distinguish between external framings (the organisation of events along the patient trajectory) and thematic framings (how clinicians and patients understood the purpose) of consultations. The way in which a participant might approach an interaction, or in our case a consultation, that is framed in a certain way can be described (again, following Goffman) as the ‘key’ for the interaction. *Rekeying* an interaction therefore involves altering its purpose or tone.

## Methods and research setting

This study was part of the OSIRIS mixed-methods research programme. Three types of surgery were chosen as offering different kinds of decisions: orthopaedic surgery for hip or knee replacement, colorectal surgery for bowel cancer and cardiac surgery. Major joint replacement is not regarded as essential in terms of life-saving but can improve quality of life (usually considered as life-enhancing). Colorectal surgery for bowel cancer is usually considered essential for survival (generally regarded as life-saving) and requires relatively rapid decisions about treatment. Cardiac surgery was offered to patients in our study for coronary artery bypass grafting (usually considered life-saving), and mitral and aortic valve repair (usually considered as life-enhancing or prolonging). Four hospital sites were recruited across two UK nations offering maximum variety in terms of diversity of population served and size/type of hospital (see Table 1). Further details are published in the study protocol (Shaw et al., 2020). The study received ethical approval from South Central Oxford C Research Ethics Committee (19/SC/0043). All research participants provided written informed consent.

**Table 1.** Summary of research sites.

Site	Size of population served*	Location of site and population	Provision of surgery selected for study
1: large teaching hospital and tertiary centre	655,000	Urban/rural	Cardiac, colorectal, orthopaedic
2: district general hospital	200,000	Urban/rural	Colorectal, orthopaedic
3: large teaching hospital and regional specialist centre	1,300,000	Major urban/urban	Cardiac, colorectal
4: large teaching hospital and regional specialist centre	2,500,000	Major urban	Cardiac, colorectal, orthopaedic

* Data from CQC and NHS Trust websites 2020.

Data were collected over 18 months during 2019-20 relating to: (1) the clinical context, (2) interactions during decision-making consultations, and (3) patient, family member and clinician accounts of decision-making processes. Clinical context data comprised 60 hours of ethnographic observations across four sites including observations of multi-disciplinary meetings, pre-operative assessment clinics, clinician training and informal interviews. Interactional data comprised video-recordings of 21 consultations between 16 patients who were considering major surgery and 9 clinicians including 8 surgeons and 1 anaesthetist (some patients had more than 1 consultation and some consultations also involved clinical nurse specialists) comprising 442 minutes of video. One researcher (GH) was present to both observe and video-record 19 of these consultations, 2 of the consultations were video-recorded with no researcher present. The clinicians we observed and interviewed were appointed between 5.5 and 21 years previously as consultants.

Interviews with patients (accompanied by family members/friends) and clinicians were conducted after consultations (immediately or within 1 week) and again with 14 of the 16 patients recruited between 3 and 11 months later (2 patients were lost to follow up). A total of 46 interviews produced 964 minutes of audio-recordings. We recruited five orthopaedic patients, five colorectal patients and six cardiac patients from three of the four sites. Patients were between 65 and 81 years old, 6 were women and 10 were men. All scored at high risk of complications following surgery, according to the Charlson Comorbidity Index (CCI), a measure of risk factors for peri-operative complications (Charlson et al., 1994). Table 2 provides a summary of patients’ characteristics, presenting problems and consultations observed (Shaw et al., 2023).

**Table 2.** Summary of patients and consultations.

ID	Site	Age (sex)	CCI*	Presenting problem	Decision making consultations observed	Attended with	Decision	Genre
*Colorectal patients (n = 5)*								
P01	1	78 (M)	5	Bowel cancer screening	1, with surgeon Cr01 + CNS (Jun-19)	Wife	Major surgery (right hemicolectomy to remove tumour/rejoin bowel)	Resolution-focused
P02	1	74 (M)	5	Anaemic	1, with surgeon Cr01+ CNS (Jun-19)	Wife	Major surgery (right hemicolectomy to remove tumour/rejoin bowel)	Resolution-focused
P05	2	79 (M)	5	Anaemic, history of stomach cancer	1, with surgeon Cr03 (Aug-19)	Wife + sister	Transanal minimally invasive surgery to remove part of rectal tumour	Deliberative
P06	2	78 (M)	8	Abnormal colon detected during scan for respiratory problems	1, with surgeon Cr03 (Sep-19)	Wife	Surveillance (colonoscopy)	Deliberative – shifting to evaluative
P14	2	65 (F)	6	Weight loss initially, pain and vomiting during consultation	2, with surgeon Cr03 (Jan-20) and anaesthetist A09 (Jan-20)	Daughter	Emergency surgery to address obstructing bowel, creating stoma	Deliberative
*Orthopaedic patients (n = 5)*								
P03	2	76 (F)	5	Hip pain and mobility (bilateral hip replacement 21 years ago)	3, with surgeon Or02 (Aug-19, Oct-19, Dec-19)	Son	Watch and wait follow-up appointments with surgeon	Evaluative
P04	2	75 (M)	4	Knee pain (replacement 13 years ago)	3, with surgeon Or02 (Aug-19, Oct-19, Dec-19)	Wife	Surgery to resurface kneecap –with option to revise joint	Evaluative
P07	1	68 (F)	3+	Knee pain (replacement 5 years ago)	1, with surgeon Or04 (Oct-19)	No	Further investigations /watch + wait	Evaluative
P08	1	70 (M)	4+	Knee pain (replacement 18 months ago)	1, with surgeon Or04 (Oct-19)	Wife	Further investigations /watch + wait	Evaluative
P09	2	81 (F)	5	Knee pain	1, with surgeon Or05 (Oct-19)	Friend	Knee replacement	Evaluative
*Cardiac patients (n = 6)*								
P10	3	74 (F)	3	Breathlessness/fatigue	1, with surgeon Ca06 (Nov-19)	Husband	Aortic valve replacement	Resolution-focused
P12	1	66 (M)	3	Chest pain	1, with surgeon Ca07 (Jan-20)	Wife	Bypass surgery	Resolution-focused
P13	1	77 (M)	3+	Aortic aneurysm detected during scan for spinal problems	1, with surgeon Ca07 (Jan-20)	Wife + daughter	Surgery not advised	Resolution-focused
P15	1	72 (M)	3	GP detected ‘murmur’	1, with surgeon Ca08 (Jan-20)	Wife	Mitral valve repair	Resolution-focused
P16	3	81 (F)	5	Aortic aneurysm detected during investigations for breathlessness, multiple myeloma diagnosed	1, with surgeon Ca06 (Jan-20)	Husband	investigations ongoing	Evaluative (lack of info)
P17	3	68 (M)	3+	Under care of cardiologist- has heart disease and PCI previously	1, with surgeon Ca06 (Jan-20)	Wife	Referral to respiratory specialist (lost to follow-up)	Resolution-focused shifting to evaluative

* Charlson Comorbidity Index score.

Analysis was iterative, focusing initially on the clinical and organisational context. First, we drew on clinical context data to map out how decision-making consultations were situated in relation to important features of the organisational and clinical context, providing us with diagrammatic summaries (Shaw et al., 2020). Second, we triangulated data from patient and clinician interviews and videos of consultations to develop narrative summaries of patient cases. Third, we synthesised maps and narratives into typical decision-making scenarios, each of which summarised the dynamics between individual patients, organisational context and the decision-making approach of surgeons. We then turned to micro-analysis of video-recordings of consultations. Analysis of consultations focused on the interaction order by organising data into ‘collections’ of extracts from consultations where patients and family members expressed decisions (Sidnell, 2013). We analysed mechanics for social interaction (how people took turns in conversations, how they used verbal and non-verbal communication strategies to progress social actions) to understand how these agreements progressed. This was particularly important for consultations where there was little explicit talk about decisions or choices. In the later phase we drew on specific dimensions of genre to analyse patterns of talk and interaction: the external or institutional framing of consultations (what consultations were understood to be about), keying and rekeying of the purpose of consultations (what happened during consultations to sustain or change their purpose) and generic resources used to achieve specific communicative ends during consultations (such as question and answer sequences and explanations) (Blum-Kulka, 2004). Finally, we synthesised findings by comparing differences and similarities between the contextual maps, patient narratives and patterns of consultations to produce three genres of consultations: resolution-focused, deliberative and evaluative (Shaw et al., 2023). Emerging analysis was refined through ongoing discussion with the OSIRIS team, including the public and patient involvement group.

## Findings

We found the framing of consultations as being about life-saving or life-enhancing procedures important in producing three different genres of consultations focused variously on: resolving problems, deliberation of options and evaluation of benefits of surgery. We have reported elsewhere on these genres (Shaw et al., 2023). Here, we focus on *how* the framing of consultations produced different genres and the consequences in terms of experiences of decision-making and degrees to which decisions were perceived as shared. In the following sections, we set out how variation in both organisational contexts (e.g. different sized hospitals) and clinical contexts (the three different surgical specialities) shaped different external and thematic framings. We include extracts from the different kinds of data and phases of analysis to demonstrate both our analytic approach across the breadth of our dataset and the ways in which decision-making was interpreted after a consultation (interview data), the unfolding process of deliberation in context (a vignette) and an example of micro-analysis during a consultation (transcript excerpt).

### Framing surgery as life-saving: Resolution-focused consultations

The framing of a consultation as being about potentially life-saving surgery was produced not by the category of the procedure itself, but by the sequencing of investigations and the sharing of knowledge between clinicians and patients about the condition and the options for treatment. The external framing of the organisation and sequencing of investigations and consultations produced a thematic framing for some cardiac and colorectal patients of their consultations as being concerned with discussing life-saving surgery. The sequencing of investigations meant that patients knew about their condition (including its severity) and the likely need for surgery before their decision-making consultations. Non-surgical treatments (e.g. endoscopic resection of tumours/polyps and percutaneous coronary intervention) had been ruled out in the course of investigations. Clinicians, patients and accompanying family members had, through processes of investigation, developed a shared understanding that surgery was necessary before the consultation with the surgeon who was expected to perform the procedure. For patients at larger hospitals, surgeons would typically meet patients for the first time when they came to discuss surgery.

Discussion of potentially curative or life-saving surgery as an optimal course of action occurred during consultations with seven cardiac and colorectal patients in this study. Surgeons typically explained the presenting problem before describing the surgical solution, explaining in detail how the surgery would proceed and quantified risks of surgery. Risks were described, including bleeding, bruising, infection, and the most serious risk of mortality – typically followed by reassurance that the proposed surgery would be major but a frequently performed and therefore routine procedure.

These consultations included relatively little time for explicit decision-making or discussion of uncertainty of outcomes, for example in relation to complications of comorbidities, or for discussions about *not* having surgery. Patients wanted to resolve their problem, for example a colorectal patient expressed his desire during the consultation with the surgeon to ‘get it sorted and get it out of the way’ – referring to the tumour found in his bowel. Despite clinicians purposefully introducing choices during the consultation, for example, in the case cited here through option-listing, some patients reported feeling they had little choice other than to continue along their trajectory towards surgery, as described by the same colorectal patient when interviewed immediately after his consultation:

‘. . .Well, I don’t think it was a decision that I made, it was a decision that the consultant has made. . .they’re the ones that are going to do the operation, they’re the ones that need to make that decision. And if you’re sensible, you take their advice, and you go along with that. . .I could have turned round and said ‘no, I don’t want any surgery - I won’t want that, we’ll just leave it and see how it goes, see what happens’. . .Well, that’s my decision, my body, but that would be silly to go down that route. . .’ (colorectal patient).

This patient indicated that, while he had autonomy (his decision, his body), he did not have a decision to make despite the listing of options by the surgeon: the nature of the problem and the likelihood of effective alternative treatment rendered other options irrelevant when the framing of the consultation was about surgery to remove the (potentially life-threatening) problem of a bowel tumour.

We observed similar resolution-focused consultations with colorectal and cardiac patients where alternatives to surgical options were discussed, but not interpreted as being viable choices by patients and their clinical teams. In one case, although surgery was not recommended because of the risk of the actual procedure, it was still understood as potentially life-saving with the consequence that the patient (and family) had to come to terms with a life-limiting diagnosis.

A shared understanding of surgery as being potentially life-saving framed consultations as resolution-focused. Patients felt they had autonomy in terms of consenting to surgery, however this framing meant that they were typically not observed as demonstrably engaging in shared decision-making in consultations, and their accounts in interviews suggest that rather they were caught up in the ‘clinical momentum’ towards surgery (Nabozny et al., 2016).

### Reframing life-saving surgery in light of co-morbidities: Deliberative consultations

Colorectal surgery for bowel cancer is usually considered as life-saving. It might therefore be expected that all consultations about such procedures would be resolution-focused. However, we found that a deliberative genre of consultation emerged with three colorectal patients in the context of their comorbidities. The uncertainty of positive outcomes for these patients led to one colorectal surgeon creating deliberative consultations, involving appraisal of a number of options.

These consultations followed similar sequences of investigation as described in the previous section, with one important contextual difference. Due to the relatively small size of the hospital site, the same surgeon was involved in the investigations (i.e. endoscopies) and the decision-making consultations. The surgeon learned about patients’ comorbidities at an earlier stage, and so advised patients to bring family members to consultations specifically designated for discussion about next steps in colorectal treatment, alongside full consideration of their general health, frailty and social circumstances. This genre of consultation resulted in more surgical options opening up as the discussion moved from the curative major surgery that would usually be considered clinically appropriate to considering options that could have a lesser impact on the patients. The vignette of Will in Table 3 shows the process of deliberation in the context of his comorbidities and his personal preferences and priorities.

**Table 3.** Vignette of Will consulting with colorectal surgeon.

Will, aged 79, lived with his wife. They had 3 children and 7 grandchildren. Since treatment for stomach cancer in his sixties, Will had been frail and underweight. A routine blood test at his GP indicated anaemia. Will was sent for investigations with a colorectal surgeon who discovered two rectal tumours. The surgeon performed a sigmoidoscopy to remove the lower tumour and invited Will to bring his wife for a consultation to decide what to do about the remaining tumour. Concerns about Will’s frailty and the impact of major surgery were shared during the consultation which included a lengthy discussion of three options; 1) do nothing, 2) remove a part of the tumour via a less invasive local procedure (TAMIS) or 3) excise the tumour via an open procedure (major surgery). The consequences of major surgery were described by the surgeon: ‘You’re going to be a higher risk patient for [major surgery] . . .if you get lots of complications . . . you might come out of hospital needing carers . . .I think the worst case scenario for you is maybe you don’t get the opportunity to go back home because you need so much support. . . or [local surgery]. . .it’s not something that’s offered to everyone but your results so far are going to suggest that’s an option. . .’ The possibility of needing long term care and, as his wife pointed out, being unable to spend time with his grandchildren, swayed Will to go for the ‘middle’ surgical option (2). This decision unfolded in discussion between Will, his wife, and the surgeon, concluding with Will’s words: ‘That’s final. Final. Made up my mind’, and the surgeon checking with Will’s spouse: ‘What do you think?’ to receive the reply: ‘I think [option] two, ‘cos I don’t think he’ll. . . . . (addressing Will) I don’t think you’ll get through [option] three’.

Will’s frailty put the usual (potentially curative) surgery in a different light. Discussion during the consultation painted a picture of high risk of complications following surgery, and the potential for loss of independence. The proposed life-saving surgery was re-interpreted, through the course of the consultation, as life-threatening (in terms of the kind of life Will wanted to lead.)

The external framing, shaped by the repeated interaction of the surgeon with the patient at both investigation stage and decision-making stage, led to a thematic framing of a consultation concerned with debating the options. The communication practices during the consultation involved identifying and discussing those options. The surgeon identified more surgical options as the discussion moved from curative major surgery to considering procedures likely to have a lesser impact. Options were jointly constructed; the surgeon offered their view of feasibility (drawing on their epistemic medical authority) and the patient and his family members talked through the possible outcomes (with a sense of shared deontic authority). Discussions concluded with mutual commitment between clinician, patient and family members to future action.

### Framing surgery as beneficial rather than necessary: Evaluative consultations

Resolution-focused and deliberative consultations were framed in broadly similar ways by clinicians, patients and family members who came to consultations with some degree of shared understanding of what the consultations would be about. Clinicians and patients had different starting points for evaluative consultations.

Evaluative consultations involved surgery understood by clinicians as potentially *beneficial* (in terms of ameliorating pain or other symptoms i.e. life-enhancing) but not *necessary* (i.e. the surgery would not be life-saving). Patients, on the other hand, were expecting resolutions to their problems. Orthopaedic consultations involved the greatest divergence between the initial framing of the consultation by clinicians (as a process of assessing whether patients might benefit from any surgery offered) and by orthopaedic patients who generally arrived at their consultation expecting surgery to resolve their symptoms – often having previously received non-surgical treatments that had failed to relieve their symptoms, and some having previously had successful joint surgery.

Surgeons knew that the desired outcomes of pain relief were never guaranteed, and so approached consultations as being concerned with evaluating whether surgery might help rather than being about deciding whether to have surgery. Surgeons rekeyed consultations away from being about the next steps towards having surgery (as patients expected) to being about whether to have surgery at all (evaluating any potential benefits of surgery), using a series of questions to explore the clinical problem and providing information about the potential risks and benefits of surgery. The extract in Table 4 from a consultation between an 81-year-old patient with knee pain and orthopaedic surgeon shows how the presenting problem was weighed up early on in the consultation in relation to other health problems and then the later shared commitment to future action, or decision.

**Table 4.** Extract from consultation between orthopaedic surgeon and patient.

Surgeon: How are you managing day to day? Patient: It’s still giving way sometimes, quite a number of times . . .the day and the pain is quite sore Surgeon: Which is worse, the giving way or the pain? Patient: Both Surgeon: Both ok. . .where you feel the pain . . .is it waking you up in the night? . . .Once you are asleep do you get woken up. . . . And what pain killers are you on? . . .Does that help? Patient: Doesn’t get rid of the pain but it helps. . . Surgeon: And how do you feel about things? Patient: I feel its holding me back stopping me doing things I used to do and erm it’s the pain Surgeon: Do you feel that that’s what’s stopping you or do you feel that the breathlessness that you were having with your heart problem that’s stopping you? Patient: It’s the pain *** Surgeon: . . .ok, how do you feel. . .given that the option would be for major surgery, for want of a better expression? Patient: I was hoping it wouldn’t come to that but at the same time if it needs doing and it going to give me less pain and a bit more mobility I would do it. Surgeon: You would go for it Patient: Yes

The first part of this extract is typical of evaluative consultations observed with all five orthopaedic patients. Clinicians asked about general problems (pain, difficulty walking), specific problems including locating pain (outside of knee, hip, back), the extent of pain (e.g. on a scale of 1–10) and how the problem affected the patient’s life (‘Do you have stairs at home?’). Some surgeons also explicitly used option grids (short decision support tools as studied by Elwyn et al., 2013) to set out risks and benefits and provided statistics about the likelihood of surgery resolving symptoms. For example, one surgeon explained that a small proportion of people who had replacement knee surgery would still experience pain even after a ‘successful’ operation.

Three consultations with cardiac and colorectal patients also took an evaluative turn when surgeons rekeyed their initial framing of the consultation (which had been to discuss surgery) to reconsidering if surgery would be beneficial when new information came to light. For example, a colorectal patient’s laboured breathing on walking into the consulting room alerted the surgeon to respiratory difficulties. Further questioning revealed recent episodes of acute illness which changed the clinician’s framing of surgery to remove a small bowel tumour as potentially life-saving to possibly life-limiting, and a subsequent mutual commitment by clinician and patient to ‘watchful waiting’ rather than surgery; this evaluative consultation involved a weighing up the benefits of having surgery at all, as opposed to deliberating about different options.

Evaluative consultations involved clinicians rekeying patients’ expectations about the possibility of surgery relieving their symptoms, for example by explaining the limitations of orthopaedic surgery, and how complications might further impede their quality of life. Rekeying also involved changing the thematic framing of consultations through interaction when the severity of patients’ co-morbidities introduced uncertainty as to whether proposed cardiac or colorectal surgery would be beneficial.

Overall, our findings showed how important organisational and clinical contexts were for the framing of consultations, with patient participation in decisions shaped by context, and consultations rekeyed by clinicians. Investigations that highlighted the seriousness of conditions led to a shared understanding between clinicians and patients that subsequent consultations were resolution-focused, concerned with discussing the next steps towards life-saving surgery, rather than making explicit decisions about whether or not to have surgery. When the clinical context of significant co-morbidities became known in advance, deliberative consultations concerned with making decisions about different surgical options were possible. A clinical context of beneficial (life-enhancing) rather than necessary (life-saving) surgery led to clinicians taking evaluative approaches, rekeying patients’ expectations about the purpose of consultations.

## Discussion

This study offers new insights into the significance of the organisational and clinical context on how consultations unfold and the role of medical authority in enabling patients to fully engage in shared decision-making during those consultations. Our identification of certain dynamic features of organisational and clinical context (the condition which led the patient to consider surgery, how that condition was understood by patient and clinicians, the organisation of services to respond to that condition, and patients’ co-morbidities) builds on Rapley’s (2008) work to show how decision-making can be distributed. These features shape the organisation of consultations (including when they take place and who is present), the matters that are discussed (options of surgery or other actions) and how those matters are discussed (as life-saving or life-enhancing) which in turn influences the next stages of the unfolding trajectory. The possibilities for patients to engage in decision-making per se appear to be reduced following a shared framing, between patients and clinicians, of a consultation as being about life-saving surgery; explicit decisions are rendered unnecessary when life-saving surgery is required. Patients can feel there is no decision to make, despite communication practices such as option-listing. The exercise of patient choice, as a decision between different options, is constrained but with little apparent conflict with medical authority due to a shared understanding of the necessity of surgery. Such resolution-focused consultations therefore culminate for the patient in exercising their autonomy by consenting to surgery rather than making a shared decision about surgery.

Medical authority is drawn from objective signs of disease; unequivocal biomedical evidence (Rasmussen, 2020) was used in our data by clinicians to rekey consultations, for example to guide patients seeking a surgical solution to considering whether they should have surgery. By establishing the objective causes of symptoms and introducing evidence of risks and benefits, clinicians can use their medical authority to open up discussion and, in so doing, dissuade some patients from major surgery. This reflects how doctors have been shown to sustain their medical authority in face of patients’ resistance (Stivers and Timmermans, 2020), for example through orienting to patients’ ‘best interests’ (Boluwaduro, 2021). Evaluative framing and communication practices can enable patients (and clinicians) to gain knowledge about likely negative outcomes of surgery, and therefore potentially reduce decision conflict and regret for high-risk patients contemplating major surgery.

The framing of a consultation as deliberative holds the greatest possibility of achieving the ideals of shared decision-making (as in Elwyn et al.’s (2014) model of collaborative deliberation). Such deliberation involves the sharing of epistemic authority (patient’s knowledge about their preferences post-surgery and surgeon’s knowledge of likely complications) to create a new contextualisation of major surgery that sets the potential consequences of a routine procedure against specific comorbidities and priorities. This can lead to a further sharing of deontic authority between clinician and patient, when the clinician relinquishes their right to determine which treatment the patient might follow. However, such consultations may only fully engage patients in shared decision-making when facilitated by the prior framing of the consultation (which owes much to organisational as well as clinical context) in addition to the communication practices used by the clinician.

Although the tensions between medical authority and patient autonomy can be arguably lessened through deliberation, clinicians’ authority persists through the framing of the consultation and their sharing of knowledge. For example, treatments that the patient might choose from are constrained by what the surgeon will offer: whilst the surgeon might avoid recommending one option over another, they will not offer an option that they have decided is too clinically risky. The problem of patients exercising full autonomy within the context of imbalances in power and knowledge between patients and clinicians remains. Efforts to promote patients’ autonomy, and an awareness of the constraints in achieving this, need to be considered as part of the broader clinical and organisational context rather than being solely reliant on communication practices during consultations.

From the patient perspective, the experience of ill-health itself and the subsequent lack of control and choice inherent to experiences of illness, ageing and dying also threatens their autonomy (Borgstrom, 2015; Schei, 2006). The autonomy patients are able to achieve in the context of considering surgery that is understood to be life-saving is therefore not only relational (produced in relation to others) but *relative* to their clinical context. The expression of self-determination for patients is a negotiated process of candidacy (Dixon-Woods et al., 2006b) for treatments throughout their trajectory; shaped by what they feel to be appropriate interventions in relation to their clinical context rather than the exercising of ‘free’ choice. Amending the clinical and organisational context which frames consultations, and altering patients’ prior perspectives on the desirable course of action, is not necessarily achievable solely through the means of improved communication practices during consultations.

### Limitations and strengths

A strength of this article is the novel analysis, informed by linguistic ethnography, of how context shapes decision-making interactions. The large dataset allowed for a combination of micro-analysis of interactions between patients, family members and clinicians and analysis of interview and contextual qualitative data. However, our findings were drawn from a relatively small number of patients and clinicians, albeit from a variety of organisational and clinical settings. Further study and analysis of a wider range of clinicians and patients would enable us to further test and extend our understanding of the importance of different kinds of contexts for shared-decision making.

## Conclusion

These findings highlight the importance of the clinical context for decision-making in producing different kinds of decision-making experiences, provide further insight into how choice relating to surgery is perceived by patients, and extend our understanding of the dynamics between medical authority and patient autonomy in the context of decision-making. Shared decision-making can increase knowledge about likely negative outcomes of surgery (which can reduce decision conflict and regret for high risk patients contemplating major surgery) and engage patients in determining courses of action. Interventions to improve shared decision-making should consider the broader context and framing of consultations, including the sequencing of events leading up to consultations and the time available for deliberation in addition to the development and use of specific communication practises during consultations.

Whilst the moral obligation to engage patients in shared decision making remains, so does the tension between offering autonomy and choice and the social context of imbalances in power and knowledge between patients and clinicians. A more moderate approach to shared decision making, which acknowledges how medical authority pervades the clinical context, could be used to overtly frame consultations as deliberative as so moderate the persistent asymmetry between clinicians and patients.
